# Response of Aquatic Organisms Communities to Global Climate Changes and Anthropogenic Impact: Evidence from Listvennichny Bay of Lake Baikal

**DOI:** 10.3390/biology10090904

**Published:** 2021-09-13

**Authors:** Lyubov Kravtsova, Svetlana Vorobyeva, Elena Naumova, Lyudmila Izhboldina, Elena Mincheva, Tatyana Potemkina, Galina Pomazkina, Elena Rodionova, Natalya Onishchuk, Mariya Sakirko, Ivan Nebesnykh, Igor Khanaev

**Affiliations:** Limnological Institute Siberian Branch of the Russian Academy of Sciences, Ulan-Batorskaya, 3, 664033 Irkutsk, Russia; lana@lin.irk.ru (S.V.); lena@lin.irk.ru (E.N.); yu_izhboldin@mail.ru (L.I.); mincheva@lin.irk.ru (E.M.); tat_pot@lin.irk.ru (T.P.); galina@lin.irk.ru (G.P.); rodionova@lin.irk.ru (E.R.); onischuk@lin.irk.ru (N.O.); sakira@lin.irk.ru (M.S.); canis-87@mail.ru (I.N.); igkhan@lin.irk.ru (I.K.)

**Keywords:** plankton, benthos, community composition, diversity, eutrophication, Lake Baikal

## Abstract

**Simple Summary:**

Lake Baikal is ranked first among the world’s lakes in terms of freshwater reserves (23,000 km^3^). It is a UNESCO World Heritage Site, and its biota is represented by unique fauna and flora, with endemics accounting for more than 60%. What is happening in the Baikal ecosystem in recent decades due to global climate change and anthropogenic impacts? In this paper, we studied this issue on the example of one of the few open bays on the western shore of Lake Baikal, as well as of some remote areas. It has been found that the plankton composition is dominated by thermophilic species; the role of endemic species in the formation of total biomass is decreasing, which confirms the ecosystem’s response to climate warming. As a result of human activity, filamentous algae bloom suppresses endemic algae species and reduces mollusk proportion. The coastal zone of Lake Baikal is taking on the features of common shallow freshwater lakes due to the predominance of cosmopolitan and widespread Palearctic species. It is necessary to monitor the Baikal ecosystem in the changing climate and to strengthen control over human activities on the shores of the lake.

**Abstract:**

Recent studies have revealed how the freshwater biota of Lake Baikal responds to climate change and anthropogenic impacts. We studied phyto- and zooplankton, as well as phyto- and zoobenthos, in the open coastal waters of the southern basin of the lake and of Listvennichny Bay. A total of 180 aquatic organism taxa were recorded. The response of the Baikal ecosystem to climate change can be traced by changes in the species composition of planktonic communities of the lake’s open coasts in summer. The key species were thermophilic the *Anabaena lemmermannii* P. Richt. (*F_ij_* = +0.7) blue-green algae, the *Asplanchna priodonta* Gosse (*F_ij_* = +0.6) rotifers in 2016, the *Rhodomonas pusilla* (Bachm.) Javorn. (*F_ij_* = +0.5) cold-loving algae, and the *Cyclops kolensis* Lilljeborg (*F_ij_* = +0.9) copepods in the past century. The proportion of Chlorophyta decreased from 63% to 17%; the Cyanophyta increased from 3% to 11% in the total biomass of phytoplankton; and the proportion of Cladocera and Rotifera increased to 26% and 11% in the biomass of zooplankton, respectively. Human activity makes an additional contribution to the eutrophication of coastal waters. The *Dinobryon* species, the cosmopolitan *Asterionella formosa* Hass. and *Fragilaria radians* Kütz., dominated phytoplankton, and filamentous algae, *Spirogyra*, dominated at the bottom in the area with anthropogenic impact. The trophic level was higher than at the unaffected background site: the saprobity index varied from 1.45 to 2.17; the ratio of eutrophic species to oligotrophic species ranged from 1:2 to 3:1, and the ratio of mesosaprobiont biomass to endemics biomass ranged from 2:1 to 7:1. Currently, the boundaries of eutrophication zones of shallow waters in Lake Baikal are expanding, and its coastal zone has acquired features typical of freshwater bodies of the eutrophic type.

## 1. Introduction

Global climate change is a key problem in the modern world. Over the past one million years, the deviation of the planet surface temperature from the maximum was about 1 °C, i.e., from 1961 to 1990 and from 2000 to 2019, the global surface temperature increased on average by 0.66 °C, and its variation in the Northern Hemisphere is higher than in the Southern Hemisphere [1]. Climate change and temperature increase are evident not only globally but also regionally, particularly in Central Asia [2,3,4]. A rise in global surface temperature affects the wind speed, precipitation regime, occurrence of drought and fires on the planet, and the rise of the water level in the World Ocean. At the same time, warming climates are transforming lake ecosystems worldwide [5,6]. For example, due to climate change, Lake Biwa has also exhibited an increase in its water temperature and a shift in its trophic status. The changes in the total phytoplankton biomass in the lake have caused structural rearrangements in the zooplankton community [7]. Climate warming and the increase in water temperature of Lake Tanganyika have intensified the stratification of the water column. This phenomenon has caused the accumulation of nutrients in deep water layers and limited their availability for aquatic organisms in nearshore habitats of the lake where they are most diverse. Consequently, populations of commercial fish and molluscs have been reduced in the lake [8]. Ancient lakes are among the best archives of past geological chronologies on the earth as well as of human activity [9]. Upon global climate change in the modern period, freshwater ecosystems exert multi-scale responses to the anthropogenic impact; they are characterised by own features of spatial and temporal variation in the biota. For example, the spread of aggressive invasive mollusc species in Lake Ohrid was associated, to a certain extent, with habitat transformation caused by elevated anthropogenic impact [10]. The significant eutrophication of Lake Victoria (blooms of blue-green algae and intensive growth of toxin-producing cyanobacteria) occurred in the middle of the 20th century due to high population densities along its shorelines, agricultural development, and industrialisation [11].

It is challenging to distinguish between the contribution from the anthropogenic activity and that made by natural/climatic factors to the changes in freshwater ecosystems. Furthermore, the regional effects of global climate change on aquatic ecosystem functions can be larger than that of local anthropogenic activity [12]. In this case, how has the unique freshwater ecosystem of oligotrophic Lake Baikal, one of the world’s deepest and ancient lakes, responded to the anthropogenic impact of global climate change? Lake Baikal is located in one of the most continental regions on the earth, in Central Asia, where the climate is strongly influenced by the Siberian High.

In this paper, we attempted to reveal the features in the recent structure of aquatic organisms communities on the basis of an analysis of autotrophic and heterotrophic components of the oligotrophic Baikal ecosystem. The model object was Listvennichny Bay. It is one of the few bays and one of the largest ones located on the west coast of Lake Baikal. Listvennichny Bay is a part of the lake that extends into the land, but it has free water exchange with the deep water of Lake Baikal. The hydrochemical and temperature regime, bottom sediment composition, and distribution of algae and invertebrates in Listvennichny Bay are typical for shallow water of the open shores of Lake Baikal [13,14]. Taking into account the large volume of Baikal’s water (23,000 km^3^) and its great depth (up to 1632 m), we hypothesised that climate change, as well as the local anthropogenic impact on the biota, are most evident in the coastal zone of the lake.

The aim of the study was to understand how climate change and anthropogenic impacts affect the communities structure of aquatic organisms in the coastal zone of Lake Baikal.

## 2. Site Description

### 2.1. Habitat Characteristics: Natural and Climatic Factors

**Figure 1:**
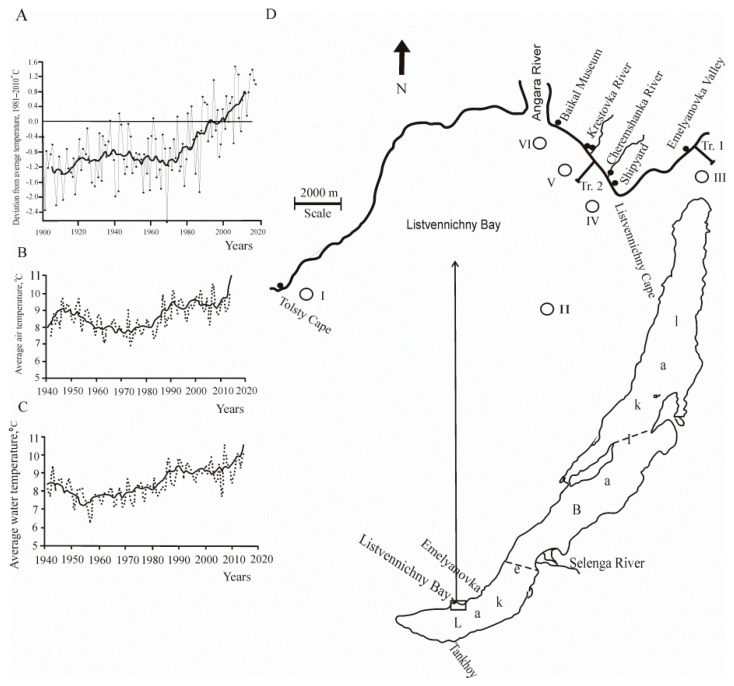
Ambient temperature trends for the territory of Russia and the Baikal region, and sampling map of Lake Baikal: (**A**) Averaged anomalies of the mean annual temperature at the earth’s surface for the territory of Russia from 1901 to 2018 (according to the Y.A. Izrael Institute for Global Climate and Ecology; the data is free available from http://climatechange.igce.ru (accessed on 6 September 2021)). (**B**) Averaged air temperatures in the Baikal region. (**C**) Averaged surface water temperatures of Lake Baikal (according to [15]). (**D**) Sampling station. Plankton sampling at two sites at depths from 0 to 15 m of the water layer. The background Site 1 included stations that are outside the zone with the impact of human activity: I—opposite Tolsty Cape in the open part of the southern basin 14 km in a southwestern direction from Listvennichny Cape (N 51°47′309; E 104°36′736); II—open part of the southern basin 7 km off the shore in direction to Listvennichny Cape-Tankhoy settlement (N 51°48′718; E 104°53′126); III—opposite Emelyanovka Valley in northwestern direction 5 km off Listvennichny Cape (N 51°51′529; E 104°56′463). Site 2 with anthropogenic impact included stations located in the coastal zone of Listvennichny Bay adjacent to the Listvyanka settlement at a distance of 50–100 m off the shore opposite the shipyard (IV) (N 51°50′479; E 104°52′772), Krestovka Valley (V) (N 51°51′236; E 104°51′595), and Baikal Museum (VI) (N 51°52′021; E 104°49′683). Benthos samples were collected from stones at the transects from the 0 to 5 m depth range opposite Emelyanovka Valley (Tr. 1) (N 51°51′529; E 104°56′463) and Krestovka Valley (Tr. 2) (N 51°51′236; E 104°51′595). Light circles are plankton sampling stations and water sampling stations for hydrochemical analysis in the Baikal coastal zone; dark circles—water sampling stations for nutrient determination in rivers and groundwater on the beach; the dotted line indicates the boundaries between the southern, central, and northern basins of Lake Baikal.

Global climate change, including the territory of Russia (according to the Institute of Global Climate and Ecology, http://climatechange.igce.ru (accessed on 6 September 2021), has become particularly evident in recent decades. In 2016, in Russia, the summer was extremely warm, with air temperature anomalies of +1.86 °C in June, +1.43 °C in July, and +2.05 °C in August. The average seasonal anomaly was +1.78 °C, the highest value since 1936 (Figure 1A).

In the Baikal region, an increase in air temperature has been observed in all seasons since the beginning of the 1970s (Figure 1B,C). Global warming has caused a rise in air temperature in the Baikal region by 1.2 °C, and water temperature in the surface layer of Lake Baikal (in May–September) has increased by 1 °C [3]. During the observation period from 1860 to 2010, the duration of ice cover has reduced by 17 days in the southern basin of Lake Baikal (Listvyanka settlement) [16]. Wind action and seasonal precipitation reduced from 2005 to 2014. The greatest anomaly of low precipitation was observed in 2015 and accounted for 52% of the norm (1961–1990) [15].

### 2.2. Anthropogenic Impact

Alongside the effects of climate change, the anthropogenic impact on the coastal waters of Lake Baikal has increased due to expanding tourism [17,18]. The Listvyanka settlement, a popular tourist destination on Lake Baikal, is located on the coast of Listvennichny Bay between the source of the Angara River and Listvennichny Cape, along valleys of the rivers that flow into Lake Baikal. Despite the growing flow of tourists in the Listvyanka settlement, with a population of about 2000 residents and numerous hotels and cafés, there is still no centralised wastewater treatment infrastructure.

The maximum concentrations of nutrients in the surface and near-bottom water layers of the coastal zone in 2011 were as follows: P-PO_4_^3^^−^ = 0.138 mg L^−1^, NH_4_^+^ = 1.898 mg L^−1^, NO_3_^−^ = 0.20 mg L^−1^, and NO_2_^−^ = 0.164 mg L^−1^. In groundwaters of the bay’s beaches, there were the following concentrations of nutrients: P-PO_4_^3^^−^ = 0.055 mg L^−1^, NH_4_^+^ = 2.333 mg L^−1^, NO_3_^−^ = 0.71 mg L^−1^, and NO_2_^−^ = 0.898 mg L^−1^ [17]. High concentrations of nutrients (NH_4_^+^ = 1.69–4.40 mg L^−^^1^, NO_2_^−^ = 0.067–0.094 mg L^−^^1^, NO_3_^−^ = 0.90–1.19 mg L^−^^1^, P-PO_4_^3^^−^ = 0.424–0.910 mg L^−^^1^, and TP (total phosphorus) = 0.696–1.298 mg L^−^^1^) were observed in 2015 in the near-bottom layers of water, in the depression topography of Listvennichny Bay, with the intense processes of decomposition of organic matter after a bloom of filamentous algae [19]. The concentration of Cl^−^, a marker of anthropogenic pollution in the shallow water of Listvennichny Bay, ranged from 1 to 4 mg L^−1^ [20] and was higher than in another area (0.45 mg L^−1^) [21]. There were also high nutrient concentrations (P-PO_4_^3^^−^ = 0.097 mg L^−1^, NH_4_^+^ = 0.040 mg L^−1^, NO_3_^−^ = 14.98 mg L^−1^, and NO_2_^−^ = 0.133 mg L^−1^) in tributaries of the bay [22]. Moreover, sanitary and microbiological maximum indicators were as follows: TC (thermotolerant coliforms) = 1000 CFU (colony forming units), *Enterococcus* = 154 CFU, and *Escherichia coli* = 1020 CFU, which also testifies to the anthropogenic impact on the nearshore zone of Lake Baikal adjacent to the Listvyanka settlement [23].

## 3. Materials and Methods

### 3.1. Field Studies

Communities of aquatic organisms in the southern basin of Lake Baikal were studied in the low-flux year, in August 2016. At this time, the waters in the coastal zone are maximally warm, and there is mass development of seasonal planktonic and benthic algae. The study area is located in the same climatic conditions. The underwater landscape of the study area extending from Kultuk to Cape Kadilny along the western side of the southern basin of Baikal. The shallow terrace both in and out of Listvennichny Bay is of abrasion type, cut into crystalline rocks of the Archean complex. Pebbles, boulders, fragments of bedrock, sand, and gravel deposits prevail in the bottom sediments. To understand the influence of natural climatic factors (without the anthropogenic component) on aquatic organisms in the coastal zone, we divided the study area into two sites: the background area (Site 1) and the area with high human activity (Site 2). The background Site 1 included stations that were outside the area with human activity impact: opposite Tolsty Cape (I) in the open part of the southern basin, 14 km to the southwest from Listvennichny Cape; in the open part of the southern basin, 7 km off the shore, in the direction of Listvennichny Cape–Tankhoy settlement (II); and opposite Emelyanovka Valley, 5 km northwest off Listvennichny Cape (III). Site 2 with anthropogenic impact included stations opposite the shipyard (IV), Krestovka Valley (V), and Baikal Museum (VI) located in the coastal zone of Listvennichny Bay adjacent to the Listvyanka settlement, at a distance of 50–100 m off the shore (Figure 1D). Numerous ships and motorboats here stand by the shore or cruise with tourists in the bay, and there are many people relaxing on the beach or engaged in scuba diving.

In 2016, the water temperature in a depth layer from 0 to 15 m at the investigated sites of Lake Baikal ranged from 15 to 17 °C. The water transparency measured with a Secchi disk was 9 m in open water (Site 1) and 7–8 m in Listvenichny Bay (Site 2).

#### Water Chemistry

To characterise the condition of the environment, we collected the surface water samples (1.5 L each) using a bucket and sampled near-bottom water from a depth of 15 m using a bathometer to determine the nutrient concentration at the study sites. Additionally, water samples were taken with a bottle at the estuaries of the Krestovka River and the Cheremshanka River as well as groundwater in the holes on the beach. A total of 31 water samples were collected. Nutrients (Si, NH_4_^+^, NO_2_^−^, NO_3_^−^, P-PO_4_^3^^−^, TDP (total dissolved phosphorus)) and TP in Baikal water samples were determined according to [24,25]; pH was measured using a pH meter (Expert pH, Russia).

The conductivity (EC_25_) of water in the Baikal coastal zone on the surface and at the bottom was from 121.3 ± 0.4 μS cm^−^^1^ to 121.5 ± 0.5 μS cm^−^^1^ (Site 1) and from 121.2 ± 0.2 μS cm^−^^1^ to 120.7 ± 0.2 μS cm^−^^1^ (Site 2), respectively; in groundwater of the beach, the conductivity was 146.0 ± 25.0 μS cm^−^^1^ (Site 1) and 200.8 ± 49.7 μS cm^−^^1^ (Site 2), and in rivers, the conductivity was 213.0 ± 118.0 μS cm^−^^1^. The pH values were 7.9 at Site 1, 8.2–8.3 at Site 2, 7.5 in groundwater of the Baikal beach, and 7.7 in rivers. At present, as performed previously [26,27], the concentrations of nutrients in the surface and near-bottom water layers of the study sites were close to the values typical for open waters of Lake Baikal in summer. Beach groundwater demonstrated the differences in the concentrations of nutrients (NO_2_^−^, P-PO_4_^3−^, TDP, and TP) at the study sites where the concentrations of the first two elements at Site 2 are an order of magnitude higher than at Site 1 (Table 1).

### 3.2. Sampling of Plankton

Plankton was collected at stations I–VI. Quantitative phytoplankton samples (1.5 L each) were collected using a bathometer in water layers of 0, 5, 10, and 15 m and fixed in the Utermel solution. Replicate (*n* = 3) zooplankton samples were collected using a Judai type 88-μm mesh plankton net (37.5-cm opening diameter) in the 0–5 m and 0–15 m water layers and fixed in 4% formalin. A total of 31 quantitative plankton samples were collected. The water transparency was measured with a Secchi disk.

### 3.3. Sampling of Benthos

Benthos was studied along two transects (Tr.). Transect 1 was located at the background Site 1 opposite Emelyanovka Valley, and transect 2 was located at Site 2 with anthropogenic impact opposite Krestovka Valley (Figure 1D). Benthos samples were collected along transects from 0 to 5 m depth by scuba divers using a frame (s = 0.16 m^2^). Stones covered with algae or macrophytes on the sand were collected from the bottom, put in sacks of strong fabric, and lifted on board the ship. The stones were then placed into a large flat-bottomed container with water. Algal samples were brushed off from stone surfaces. The samples were poured through a sweep net (gauze sieve nos. 35). One-eighth of the algal sample was taken for analysis of microphytobenthos. The algae were put in separate bottles and fixed in 4% formalin. To characterise the structure of benthic phytocenoses, we collected a total of 30 quantitative samples from these transects.

At the same transects, scuba divers collected quantitative macrozoobenthos samples (14) from rocky substrates using a frame (s = 0.16 m^2^) at depths from 0 to 5 m.

### 3.4. Laboratory Analyses

Phytoplankton samples were precipitated to a 15–20 mL volume for at least 14 days. The algae were counted in a volume of 0.1 mL. Individual volumes of cells were taken into account to determine the algal biomass (mg) [28]. Zooplankton samples were counted following zooplankton counting techniques [14]. The biomass (mg) of planktonic organisms was represented per 1 m^3^ of water. Additionally, the abundance of zooplankton was counted (thousand specimens per m^3^).

Benthic microalgae with size ≤2 mm were sorted into taxa under an Axiovert-200 light microscope; the cells were counted in 0.1 mL using a Nageotte chamber (*n* = 3). The biomass (mg) was assessed using a procedure described previously [28].

Benthic macroalgae (meyo- and macrophytes with size ≥2 mm) and macroinvertebrates (≥2 mm) were sorted into supraspecific taxa using an MBS 10 microscope (LZOS, Lytkarino, Russia) at 2 × 8 magnification. Algae were identified to the species level on temporary slides with an Amplival microscope (Carl Zeiss, Jena, Germany) at 12 × 10 and 12 × 40 magnifications.

The biomass (mg) of benthic macroalgae and invertebrates was determined separately. The specimens were dried on filter paper and weighed using a VT-500 torsion balance (precision ±1 mg). Quantitative values (biomass) of benthic aquatic organisms are expressed per 1 m^2^ of the lake’s bottom.

The current communities of aquatic organisms were compared to those of previous years using archival data from quantitative collections made by S. Vorobyova (7 samples of phytoplankton in 1992), E. Naumova (6 samples of zooplankton in 1998), L. Izhboldina (6 samples of meyo- and macrophytes in 1987), G. Pomazkina (6 samples of microphytobenthos in 1998), and L. Kravtsova (15 samples of macrozoobenthos in 1988). These samples were used as reference data (RD) because they were obtained during the period characterised by low anomalies of average air temperature and other natural and climatic factors as well as by a lower anthropogenic impact on the shorelines of Lake Baikal. All samples were collected in the southern basin of Lake Baikal in July and August using the same methods as in 2016.

### 3.5. Data Analyses

All estimations were carried out using average values of aquatic organism biomass and nutrients (±standard error of the mean).

#### 3.5.1. Communities of Aquatic Organisms

To analyse the community structure, we preliminary combined the data on the biomass of aquatic plankton organisms from different water layers into one integral sample of a water layer from 0 to 15 m to calculate the average biomass at each station. The communities of aquatic organisms were distinguished according to the dominant species in terms of biomass. This indicator shows less variability compared to abundance. Clustering techniques (Ward’s method and Euclidean distances) were used to characterise the structure of aquatic organism communities by dominant taxa. Taxa with biomass less than 10 mg m^−3^ (plankton), benthic macroalgae (10 × 10^3^ mg m^−2^), and microalgae (1 mg m^−2^) were not used for calculations.

Principal component analyses (PCA) were used to determine how dominant taxa of communities are distributed at the study sites (to visualise the spatial distribution of dominant species). For this purpose, clustering results were plotted along the first two axes of an ordination diagram on the basis of the results of the PCA to assess the confinement of species to the study sites. Biomass of dominant taxa in RD, Site 1, and Site 2 were used as variables. Before that, the source data for multidimensional analysis were transformed using the log_10_ (X + 1) function.

Key species in communities of aquatic organisms at the study sites were determined by dominant species, which had the maximum positive values of *F_ij_*, the index of relative biotope confinement [29]:

*F_ij_* = (*n_ij_* × *N* − *n_i_* × *N_j_*)/(*n_ij_* × *N* + *n_i_* × *N_j_* − 2 × *n_ij_* × *N_j_*),

where *n_ij_* is the biomass of *i*-species in the *j*-th sample of volume *N_j_*, and *n_i_* is the biomass of specimens of this species in all collections of volume *N*.

The *F_ij_* value varies from −1 (absent in the habitat) to +1 (the species is present in the habitat). A zero *F_ij_* value indicates an indifferent attitude of the species to the habitat, *F_ij_* < 0 characterises weak habitat confinement of the species, and *F_ij_* > 0 indicates the presence of habitat confinement of the species.

#### 3.5.2. Assessment of Environmental Quality of the Studied Sites According to Biological Indicators

To characterise the environment at the study sites, we used biomass of all identified taxa for calculating the following indicators: the Shannon diversity index (H) to estimate *β*-diversity [30], the ratio of eutrophic to oligotrophic species counts (E/O), and the ratio of mesosaprobiont biomass to the biomass of Baikal endemics (M/E), which we proposed for the first time. Moreover, the state of the zooplankton community was assessed using k-dominance curves, in which it is under unstressed (if the biomass curve lies above the abundance curve), moderately stressed (the biomass and abundance curves intersect), or heavily stressed (the abundance curve lies above the biomass curve) conditions [31].

The trophic level of the studied areas was assessed by the saprobity index. The saprobity index was proposed by Pantle and Buck [32] and modified by Sládecek [33]:

*S* = ∑*s_i_* × *h_i_*/∑*h_i_*,

where *S* is the saprobity index of a water body or its part, *s_i_* is the saprobity value of an indicator species, and *h_i_* is the estimated frequency of saprobiont occurrence in samples. The value of the saprobity index for each indicator species (*s_i_*) was determined on the basis of the list of saprobic organisms [34,35].

The saprobity index ranges from 0.50 to 1.50 for the oligosaprobic zone, from 1.51 to 2.50 for the *β*-mesosaprobic zone, from 2.51 to 3.50 for the *α*-mesosaprobic zone, and from 3.51 to 4.50 for the polysaprobic zone.

## 4. Results

### 4.1. Biodiversity Pattern: The Species Richness and Structure of Aquatic Organisms Communities

The diversity of aquatic organisms was high at the investigated sites; a total of 180 taxa were registered.

**Figure 2:**
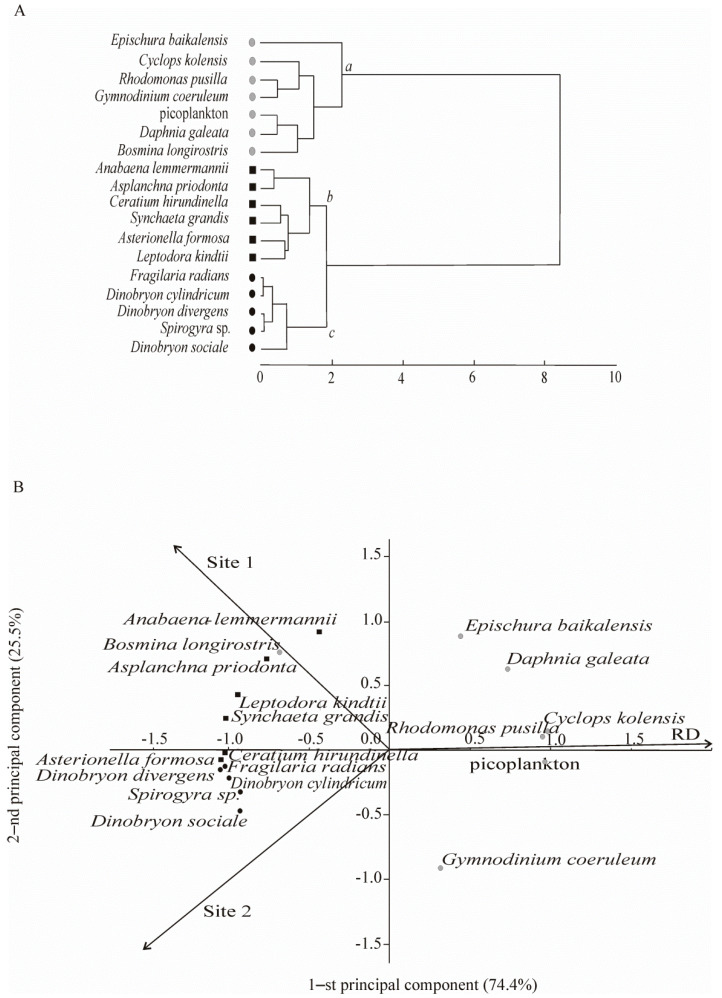
Plankton community structure and spatial distribution of dominant species in the coastal zone of Lake Baikal. (**A**) Species composition of plankton communities (*a*, *b*, and *c*) extracted by Ward’s cluster analysis using Euclidean distances. (**B**) Locations of the identified plankton communities in the plane of the first two principal components (PCA). RD—reference data, including samples collected in the southern basin of Lake Baikal in the past century; Site 1 included stations I, II, and III of the background area in 2016; Site 2 included stations IV, V, and VI of the area with human activity in 2016 (see Figure 1D).

Ninety-two taxa were found in the plankton, including such algae as Chlorophyta (18), Bacillariophyta (16), Cyanophyta (8), Ochrophyta (7), Miozoa (4), Cryptophyta (2) and Charophyta (1), as well as zooplankton invertebrates of Rotifera (20), Cladocera (10) and Copepoda (6). Eighteen taxa planktonic aquatic organisms dominated, accounting for >80% (phytoplankton) and >90% (zooplankton) of the total biomass and forming three communities (*a*, *b*, and *c*) on the basis of cluster analysis (Figure 2A).

There was spatial differentiation of the identified communities in the plane of the first two principal components. The first principal component characterised the differences between communities recorded in 2016 and communities recorded at the end of the past century (RD) (Figure 2B). In the past century, most species from community *a* occurred. The second principal component characterised the differences between community *b* (most species of this community were confined to the background Site 1) and community *c* (most species of the community were confined to the area with anthropogenic impact, Site 2) (Figure 2B).

**Figure 3:**
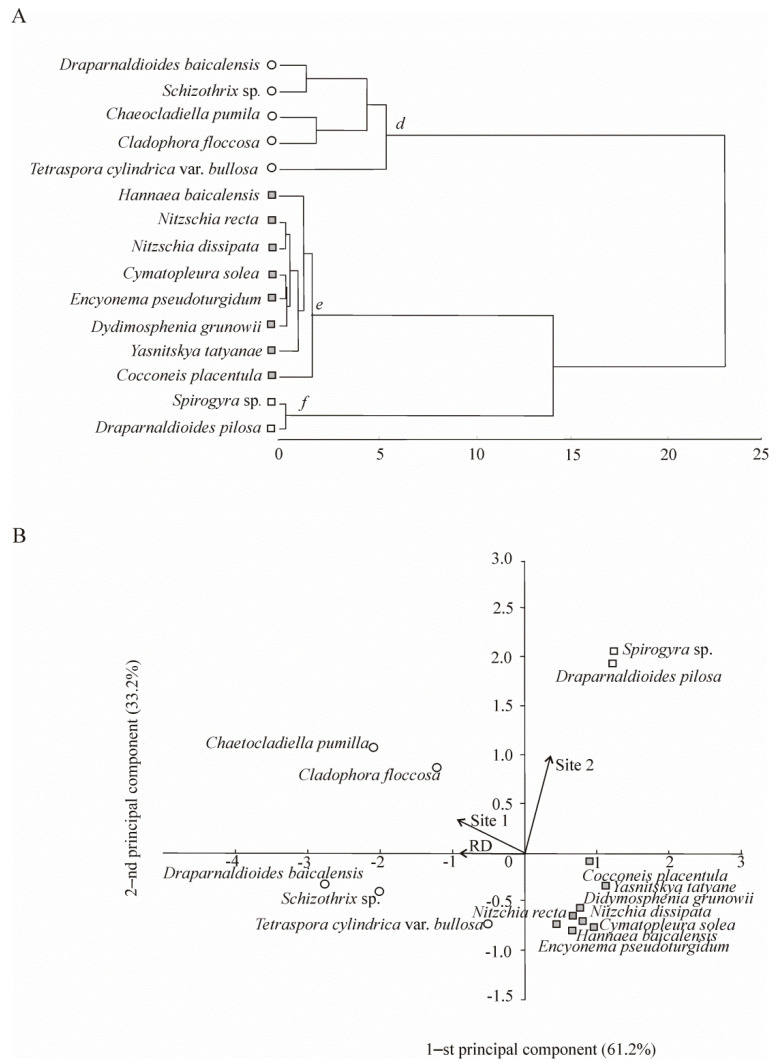
Benthic community structure and spatial distribution of dominant species in the coastal zone of Lake Baikal. (**A**) Species composition of benthic communities (*d*, *e*, and *f*) extracted by Ward’s cluster analysis using Euclidean distances. (**B**) Locations of the identified benthic communities in the plane of the first two principal components (PCA). RD—reference data, including samples collected on the stones from 0 to 5 m depth range in north-western direction 5 and 17 km off Listvennichny Cape in the past century; Site 1 included stations at transect 1 of the background area in 2016; Site 2 included stations at transect 2 of the area with human activity in 2016 (see Figure 1D).

Eighty-eight taxa were found in benthic flora, including 30 macroalgae of Chlorophyta (17), Cyanophyta (8), Charophyta (4), and Ochrophyta (1), as well as 52 microalgae of Bacillariophyta, and 6 submerged macrophytes. Fifteen taxa of the benthic algal flora dominated, accounting for >80% (macroalgae) and >57% (microalgae) of the total biomass and forming three communities (*d*, *e*, and *f*) on the basis of cluster analysis (Figure 3A).

The location of dominant benthic algal species in the plane of the first two principal components indicated that most species of community *d* occurred in the background area (Site 1) and earlier (RD); species of community *f* were confined to the anthropogenic impact area (Site 2) by the first principal components. The second principal components showed that the community of microalgae (*e*) was not confined to the studied habitats (RD, Site 1, and Site 2) and was of an uncertain nature. Apparently, the development of microalgae was not closely related to the abundance of one or another species of macroalgae, but it was determined by other factors that we did not take into account.

There were representatives of different phyla of benthic macroinvertebrates: Platyhelminthes, Annelida (Hirudinea, Polychaeta, and Oligochaeta), Arthropoda (Isopoda, Amphipoda, Trichoptera, and Chironomidae), and Mollusca, which are common inhabitants of Lake Baikal.

### 4.2. Species Composition and Diversity of Aquatic Organisms in the Background Area (Site 1)

In the background area (Site 1), in 2016, the dominant species in the community accounted for 83% of the total phytoplankton biomass (186.9 ± 22.5 mg m^−3^). The key species in the phytoplankton community was *Anabaena lemmermannii* P. Richt. (17.3 ± 4.9 mg m^−3^), with *F_ij_* = +0.7. Previously (RD), dominant species also accounted for 88% of the total phytoplankton biomass (190.7 ± 41.5 mg m^−3^), but other species, *Rhodomonas pusilla* (Bachm.) Javorn. (22.9 ± 6.9 mg m^−3^) and picoplankton (117.4 ± 39.4 mg m^−3^), played a key role. Their *F_ij_* indexes were +0.5 and +0.7, respectively (Figure 2, Table 2).

The total number of phytoplankton taxa (45) in 2016 was close to RD (42), while the species *β*-diversity, according to the Shannon index, increased (Table 3). This area was an oligosaprobic zone in 1992 (*S* = 1.47) and transformed into a *β*-mesosaprobic zone in 2016 (*S* = 1.60). The ratio of the mesosaprobiont (flagellates) biomass to the endemic biomass shifted towards an increase in the mesosaprobiont biomass (Table 3).

**Figure 4:**
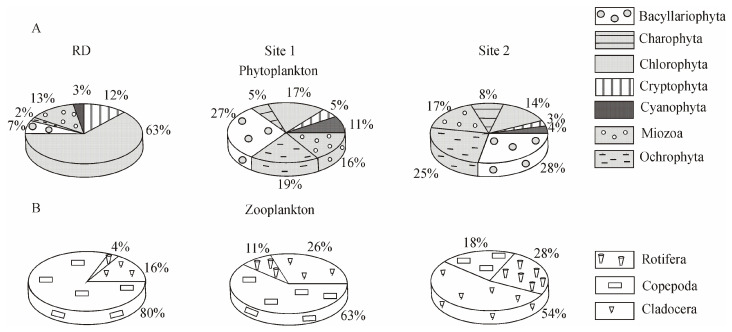
The proportion of higher taxa in the formation of the total plankton biomass in the studied areas of the Baikal coastal zone. (**A**) Percentage ratio of biomass of phytoplankton higher taxa; (**B**) percentage ratio of biomass of zooplankton higher taxa. RD—reference data, including samples collected in the southern basin of Lake Baikal in the past century; Site 1 included stations I, II, and III of the background area in 2016; Site 2 included stations IV, V, and VI of the area with human activity in 2016 (see Figure 1D).

Shifts in the structure of the phytoplankton community occurring on the basis of the total biomass were detected at the level of higher taxa. In 2016, the percentage of Bacillariophyta, Cyanophyta, and Ochrophyta increased in the total biomass, whereas that of Chlorophyta and Cryptophyta decreased considerably compared with RD (Figure 4A).

**Figure 5:**
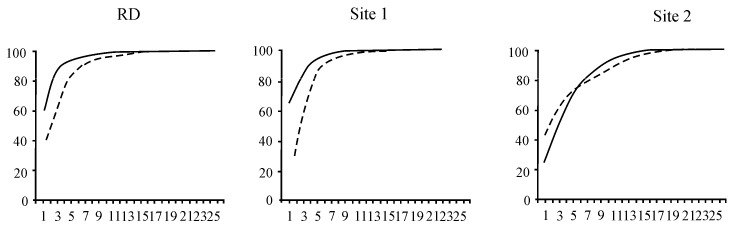
The k-dominance curves characterising the state of the zooplankton community in the coastal zone of Lake Baikal: RD—zooplankton community characterised as stable in the early period; Site 1—zooplankton community characterised as stable in the background area; Site 2—zooplankton community characterised as moderately disturbed in the area with human activity. The ordinate axis is the cumulative % of biomass (1) and abundance (2) of species, and the abscissa axis is the sequence of zooplankton species ranked in descending order.

The zooplankton community in 2016 differed from that observed during previous years of the study. In the background area, the dominant species in the community accounted for 98% of the total zooplankton biomass (723.7 ± 166.1 mg m^−3^). Besides *Epischura baikalensis* Sars (448.9 ± 175.3 mg m^−3^), *Leptodora kindtii* (Focke) (44.8 ± 22.5 mg m^−3^), and *Asplanchna priodonta* Gosse (14.6 ± 6.3 mg m^−3^) dominated zooplankton and had a high index of biotope confinement (+0.7 and +0.6, respectively) to the background area (Site 1) (Table 2). Previously (RD), the dominant species in the community accounted for 96% of the total zooplankton biomass (672.7 ± 158.6 mg m^−3^). *Cyclops kolensis* Lilljeb. (124.5 ± 76.2 mg m^−3^) dominated the zooplankton community (together with *E. baikalensis*—414.6 ± 112.2 mg m^−3^), and it played a key role in zooplankton (*F_ij_* = +0.9) (Figure 2, Table 2). At present, the role of *C. kolensis* (5.7 ± 2.2 mg m^−3^) in zooplankton has significantly decreased at Site 1; however, according to k-dominance curves, the community has remained stable and unstressed, unlike how it was previously (Figure 5).

The total number of zooplankton taxa (19) in 2016 was relatively low in the background area; the Shannon index measuring *β*-diversity varied insignificantly (1.2–1.4) over different years. The trophic level (based on the S, E/O, and M/E indicators) was almost similar to RD (Table 3). The background area (Site 1) could be classified as an oligotrophic zone on the basis of zooplankton, as it had been previously.

Copepoda and Cladocera predominated in the total biomass of zooplankton, as was the case previously; only an insignificant decrease in the proportion of Copepoda and an increase in the proportion of Cladocera and Rotifera were observed (Figure 4B).

The benthic microalgal communities were almost the same at the end of the past century (RD) and currently (Site 1) (Figure 3). In 2016, the microalgal biomass in the background area was 10.0 ± 3.2 mg m^−2^. Among them, *Cocconeis placentula* Ehrenb. (4.4 ± 1.6 mg m^−2^) dominated. Previously (RD), the microalgal biomass was higher (39.2 ± 10.5 mg m^−2^) and dominated by *Hannaea baicalensis* Genk., Popovsk. and Kulik. (14.0 ± 10.5 mg m^−2^) and *Nitzschia recta* Hantzsch. (3.7 ± 1.2 mg m^−2^), typical inhabitants of the rocky littoral zone of Lake Baikal. These species are among the key taxa of microalgae, according to *F_ij_* (Table 2). The trophic status of the background area in terms of microalgae was characterised as an oligotrophic zone (Table 3).

The total biomass of meyo- and macrophytes accounted for 245.5 × 10^3^ ± 65.5 × 10^3^ mg m^−2^ (RD) and 185.3 × 10^3^ ± 74.9 × 10^3^ mg m^−2^ (Site 1). The dominant species in the communities accounted for 88% and 96% of the total macroalgal biomass. Endemic species, *Chaetocladiella pumila* (Meyer) Meyer et Skabitsch. (RD—35.7 × 10^3^ ± 21.1×10^3^ mg m^−2^; Site 1—18.1 × 10^3^ ± 8.0 × 10^3^ mg m^−2^) and *Draparnaldioides baicalensis* Meyer et Skabitsch. (RD—123.3 × 10^3^ ± 56.0 × 10^3^ mg m^−2^; Site 1—132.8 × 10^3^ ± 71.6 × 10^3^ mg m^−2^) dominated and had higher *F_ij_* values (Table 2). Previously (RD), in addition to these species, *Cladophora floccosa* C. Meyer, *Tetraspora cylindrica* var. *bullosa* C. Meyer, and *Schizothrix* sp. also dominated and played a key role in the benthic community (Figure 3).

The total number of macroalgal species varied from 15 to 23 in different years; the Shannon index of *β*-diversity ranged from 1.0 to 1.8. There was an insignificant increase in the saprobity index (*S* = 1.57) compared to RD (*S* = 1.48), whereas the E/O and M/E ratios were stable; endemic species prevailed in the biomass (Table 3).

**Figure 6:**
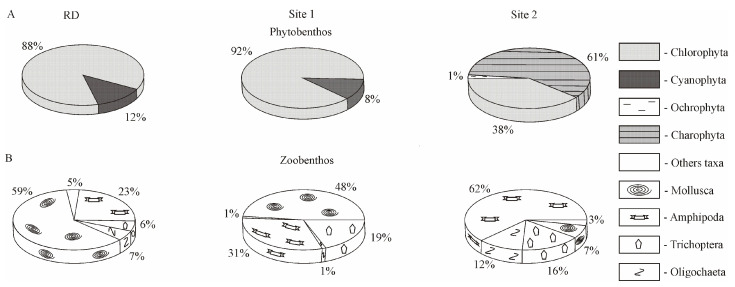
The proportion of higher taxa in the formation of the total benthic biomass in the studied areas of the Baikal coastal zone. (**A**) Percentage ratio of the biomass of macroalgae higher taxa; (**B**) percentage ratio of the biomass of macrozoobenthos higher taxa. RD—reference data include samples collected on the stones from 0 to 5 m depth range in the north-western direction 5 and 17 km off Listvennichny Cape in the past century. Site 1 included stations at transect 1 of the background area in 2016; site 2 included stations at transect 2 of the area with human activity in 2016 (see Figure 1D).

Overall, macroalgal biomass was formed mainly by Chlorophyta (88%) and Cyanophyta (12%), which is consistent with RD (Figure 6A).

Although the quantitative values of macrozoobenthos in 2016 (total biomass 17.7 × 10^3^ ± 3.2 × 10^3^ mg m^−2^) were lower compared to RD (total biomass 45.3 × 10^3^ ± 13.8 × 10^3^ mg m^−2^), molluscs and amphipods dominated the macroinvertebrates, as they had previously (Figure 6B).

### 4.3. Species Composition and Diversity of Aquatic Organisms in the Area with Human Activity in Listvennichny Bay (Site 2)

In 2016, in the area with human activity (Site 2), the dominant species in the community accounted for 86% of the total phytoplankton biomass (246.0 ± 16.3 mg m^−3^). The key species at Site 2 were *Dinobryon sociale* Ehr. (12.8 ± 2.9 mg m^−3^) and *Spirogyra* sp. (20.6 ± 7.1 mg m^−3^); their *F_ij_* were +0.7 and +0.6, respectively (Figure 2, Table 2). The latter species is representative of the benthic flora that has been forming algal mats in this area since 2011. Other dominants, namely, *Ceratium hirundinella* (O. Müll.) Schrank., *Dinobryon cylindricum* Imhof, *Dinobryon divergens* Imhof, *Asterionella formosa* Hass., and *Fragilaria radians* Kütz. (= *Synedra acus* subsp. *radians* (Küts.) Skabitsch.), are typical inhabitants of Lake Baikal in the summer. According to the indices characterising the trophic level of phytoplankton (*S* = 1.70, M/E = 6:1), the area with human activity could be classified as a *β*-mesosaprobic zone (Table 3).

The area with human activity could also be considered a *β*-mesosaprobic zone on the basis of the indicators of zooplankton (S = 1.53, M/E = 2:1). In this area, the total zooplankton biomass (100.7 ± 14.3 mg m^−3^) was lower than in the background area and according to RD (Table 3). The dominant species in the community accounted for 94% of the total zooplankton biomass. *Synchaeta grandis* Zach. (21.0 ± 4.6 mg m^−3^) played a key role at this site, with *F_ij_* = +0.7, despite the fact that this species had high biomass in the background area (Site 1) (Figure 2, Table 2). The role of *C. kolensis* (4.0 ± 1.0 mg m^−3^) in the formation of the total zooplankton biomass was also lower than RD. According to the k-dominance curves, the zooplankton community structure was less stable, i.e., it was in a moderately stressed state (Figure 5). Significant changes were especially evident in the ratio of higher taxa: compared to RD, the proportion of Copepoda decreased from 80% to 18% in the total biomass, and Cladocera increased from 16% to 54% (Figure 4B).

Among benthic microalgae, no permutations in composition were found; the same species as in the background site dominated (Figure 3B), but their biomass was lower. Although the saprobity index was 1.45, the M/E ratio of 7:1 changed towards an increase in mesosaprobiont biomass (Table 3).

In contrast to the background area and RD, the community structure of benthic macroalgae changed in 2016 due to alterations in the dominant species. The total macroalgal biomass (367.6 × 10^3^ ± 119.4 × 10^3^ mg m^−2^) was dominated by algae of the genus *Spirogyra* (94.8 × 10^3^ ± 67.0 × 10^3^ mg m^−2^), atypical species for the open coastal zone of Lake Baikal. *Draparnaldioides pilosa* C. Meyer et Skbitsch. (58.4 × 10^3^ ± 5.6 × 10^3^ mg m^−2^) also dominated and played a key role in the phytobenthos (*F_ij_* = +1), along with *Spirogyra* (*F_ij_* = +1) (Figure 3, Table 2).

The Shannon *β*-diversity index (2.1) and the saprobity index (*S* = 2.17) increased. The E/O and M/E ratios also changed compared to the background area and RD (Table 3).

The same groups of macroinvertebrates predominated in the area with human activity, but with lower total biomass (5.4 × 10^3^ ± 2.8 × 10^3^ mg m^−2^) than in the background area. Furthermore, the proportion of Amphipoda increased to 62%, Oligochaeta increased to 12%, and Mollusca decreased to 7% compared to RD at 23%, 7%, and 59%, respectively (Figure 6B).

## 5. Discussion

### 5.1. Response of Aquatic Organisms Communities to Climate Change

Climate warming can change species composition and energy flow in aquatic ecosystems. Globally, lake water temperatures (based on analysis of 102 lakes, including Baikal) have warmed rapidly relative to air temperatures, with significant increases in lake surface water temperatures found at an average rate of +0.37 °C decade^−1^, but trends in deep-water temperatures have shown little change [5]. In this regard, we focused our study on the species composition of aquatic organisms communities in the water column and at the bottom in the coastal zone of Lake Baikal, which is well heated in summer.

Global climate warming and water temperature rise in lakes such as Balaton, Tahoe, and Kinneret contributed to explosive growth of nanoplankton (size <20 µm) and increased the proportion of small-sized algae (size <2 µm) [36,37,38]; this phenomenon was also observed in Lake Baikal [6,39]. In contrast to the increasing temperature in the Baikal surface water since the early 1970s (Figure 1C), the duration of ice cover has reduced [4]; warm-loving species have replaced cold-loving ones in recent decades. The abundance of the *Aulacoseira* species during the under-ice period (April–May) from 1951 to 1999 was stable, but the average annual abundance of *Aulacoseira* has recently decreased by more than 70% [40]. Another species, *F. radians*, which predominated in the springs of 2008, 2009, 2011, and 2016 [39,41], become dominant in the summer coastal phytoplankton in 2016 (Figure 2) due to the extension of subglacial blooms. Moreover, at present time, the biomass of a typical representative of summer phytoplankton, the cold-loving species *R. pusilla* [6], was less than the biomass of the thermophilic *A. lemmermannii* in the background area. This phenomenon is an evident response of the Baikal ecosystem to global warming. These blue-green algae are vegetating in the areas of open Baikal adjacent to shallow waters during the greatest water warming [42]. After blooming, the seed bank of diatoms (44%) is deposited exactly in the coastal zone of sea and fresh waters, which serves as a depository for their reproduction [43]. Hence, the trend of the predominance of warm-loving species in Lake Baikal, including the *A. lemmermannii* blue-green algae, will be stable in time if the trend of water temperature growth remains unchanged.

Eutrophication processes in the coastal zone of Lake Baikal can occur much faster than in the deep-water zone due to the warming of the surface waters during summer stratification and the weakening of the wind regime [15]. The weakening of the wind regime in the Baikal region reflects the general trend observed in the area between 35° and 75° N, where the kinetic energy of winds decreased in the summer by 8–15% from 1979 to 2013 [44]. Due to the recent increase in stratification and reduced mixing in Lake Baikal, diatom species occupy deeper depths [45]; the same processes were observed in Lake Tahoe [37]. However, we assume that nutrient influx to the deeper layers of the photic zone plays a major role for algae. It was shown that in response to changes in natural and climatic factors, the inflow of nutrients from the deep waters of Lake Baikal into the photic zone has increased since the mid-19th century [46]. Perhaps, therefore, in summer, the cosmopolitan species *A. lemmermannii*, *A. formosa*, and *F. radians* prevail in the warm surface waters of Lake Baikal (Figure 2). The endemic species *Lindavia minuta* (Skvortsov) T.Nakov & al., and *Aulacoseira baicalensis* (Meyer) Simon., occur rarely in cold waters of deeper layers of the photic zone, where the influx of nutrients takes place in summer. The latter two species were found to be always few as well as early and had insignificant biomass <0.5% in 2016, and 2–4% in 1992.

The influx of nutrients from the watershed is very important for the Baikal ecosystem. In summer (Table 1), the concentrations of nutrients in the water of the background Site 1 remained similar to that in water along the open coastal areas of the southern basin of Lake Baikal: NO_3_^−^ = 0.3–0.4 mg L^−1^, P-PO_4_^3^^−^ = 0.005–0.010 mg L^−1^, and TP = 0.006–0.010 mg L^−1^ [21]. Despite changes in the composition of the coastal phytoplankton community, the chlorophyll *a* concentration in the southern basin of Lake Baikal near the west coast is still low (1.1–2.9 µmol L^−1^); the nutrient concentration of TN ranges from 0.05 to 0.15 mg L^−1^, TP from 0.006 to 0.024 mg L^−1^, and TN:TP (ratio for the surface water in different sites) from 7 to 38 [47]. Although the maximum values of TP (0.024 mg L^−1^) indicate the presence of mesotrophic conditions [48], in most cases, there were low concentrations of nutrients due to its intensive absorption by algal blooms in spring and benthic algae vegetation, the mass development of which occurs in summer.

In contrast to phytoplankton, the structure of benthic algal communities has remained unchanged in most areas of Lake Baikal, including the background site, and endemic macroalgae, *D. baicalensis* and *C. pumila*, and microalgae, *C. placentula* and *Didymosphenia geminata* (Lyngbye) Schmidt., dominate [49,50]. However, of note is an increase in the diversity of the benthic flora on the rocky substrates of the open shores of Lake Baikal compared to that at the end of the past century. Not only the *Spirogyra* algae but also *Oedogonium* have become frequent in the benthic algal flora [51].

On the basis of the zooplankton data, there has been an eutrophication in the shallow waters of Lake Baikal upon climate change. Warm-loving species, *A. priodonta, L. kindtii*, and *S. grandis*, the key species of the zooplankton community in the studied areas, are typical representatives of the Baikal coastal zone in summer. Currently, while *E. baikalensis* has remained stably abundant in the summer plankton, the biomass of *C. kolensis* in the background area has decreased. *C. kolensis* is known to be a cold-loving species and is not found in plankton during the maximum warming of water in the summer [14]. The decrease in the *C. kolensis* abundance may have contributed to a certain extent to an increase in the proportion of Rotifera in the total zooplankton biomass (Figure 4B). Nonetheless, the effect of temperature on the development of Rotifera is not excluded because, according to experimental studies, temperatures above 15 °C intensify the reproduction of Rotifera [52]. A rise in the proportion of Cladocera and Rotifera in the total biomass of zooplankton in 2016 (Figure 4B) agrees with the multivariate autoregressive (MAR) model that correlates the development of certain groups of zooplankton to temperature trends [53].

Due to climate warming, there is currently an increase in the proportion of Cladocera and Rotifera in the total biomass of zooplankton as well as an in increase Cyanophyta and a decrease in Chlorophyta in the total biomass of phytoplankton (Figure 4). These aquatic organisms are markers of freshwater eutrophication processes. It is likely that the change in the ratio of their proportion in the total biomass is due to their food strategy caused by the abundance of food (bacteria and detritus). The decrease in the proportion of Chlorophyta in plankton in 2016 may have been due to their consumption by rotifers. In particular, the *A. priodonta* rotifers are considered predators, but, according to [54], they can also be phytophagous in Lake Windsborn, consuming chlorococcal algae (*Pediastrum*, *Scenedesmus*, and *Ankistrodesmus*) and the *Gymnodinium* dinoflagellates.

In contrast to plankton, heterotrophs from the open coastal zone of Lake Baikal, namely, benthic macroinvertebrate communities (at the level of high taxa), have not yet shown a response to climate warming; as reported previously [13], in the 0–5 m depth zone, molluscs and amphipods prevailed (Figure 6B). However, changes in the species composition of macroinvertebrate communities are not excluded.

Unfortunately, at this stage of the study, we could not assess trophic relationships between the identified planktonic and benthic taxa due to limited data on the biology of most species, which is very important for understanding ecosystem functioning under conditions of climate change and anthropogenic impact.

### 5.2. Current Structure of Aquatic Organisms Communities in the Area with Human Activity in Listvennichny Bay

Many researchers previously considered Lake Baikal oligotrophic, functioning more similar to marine and oceanic ecosystems [55] than to freshwater lakes such as Victoria, Malawi [56], and Biwa [57]. Listvennichny Bay also represented an oligotrophic area of Lake Baikal in the past century [13]. Over the later decades, the appearance of the Baikal coastal zone has changed considerably due to human activities. Alongside the effects of global natural and climatic changes, the anthropogenic component has made an additional contribution to the eutrophication of the coastal zone of Lake Baikal. In the area subject to anthropogenic impact, there was an increase in nutrients in the groundwaters of beaches and in the coastal zone of the lake [20,21]. Wastewater from human settlements enters the coastal zone of Lake Baikal due to poorly functioning wastewater treatment facilities [18,58]. The Baikal region has recently seen a threefold increase in tourist traffic [58]. The transition from oligotrophic to *β*-mesosaprobic ecological status in Listvennichny Bay may indicate a response of the Baikal ecosystem to climate warming and anthropogenic impact. At present, we recorded higher values of saprobity index in the 0–15 m layer of Listvennichny Bay, which was accompanied by a reduced role of endemic species in the formation of the total biomass compared to the background site (Table 3). Typical Baikal species of the genus *Dinobryon* and cosmopolitan species, *A. formosa* and *F. radians*, dominated phytoplankton in the area with human activity, but the key species were *D. sociale* and *Spirogyra* (Figure 3). The latter species is a mass of algae that develops at the bottom of the bay at depths of >3 m. Overgrowth of the bottom with the filamentous *Spirogyra* algae (the algal thallus is represented by unbranched filaments) has effects on communities in the water column. Its cells have hydraulic characteristics similar to planktonic algae and can enhance autotrophic productivity in the surface water of the Baikal ecosystem [51]. In benthic communities of Listvennichny Bay, filamentous algae inhabiting a wide range of aquatic environments replaced endemic species dominant in the biomass. Overgrowth of the bottom with filamentous algae primarily indicates anthropogenic impact and the nutrient influx to the coastal waters [59,60,61,62]. The formation of filamentous algal mats at the bottom of the lake influences the distribution of macroinvertebrate communities. Surfaces covered with filamentous algae prevent the free movement of molluscs that use rocky substrates as a habitat and site for attachment of clutches. Thus, in 2016, we observed a reduction in the proportion of molluscs in the total macrozoobenthos biomass in Listvennichny Bay (Figure 6B). On the contrary, the role of oligochaetes increased because filamentous algae create a favourable habitat for these aquatic organisms, serving as a shelter and a source of food [63]. Bottom overgrowth by filamentous algae in Listvennichny Bay led to catastrophic degradation of sponge colonies [19], including the endemic *Lubomirskia baicalensis* (Pallas), which plays a major role in the self-purification of Baikal waters.

Taking into account that the response of aquatic organisms to nutrient loading and climate warming may be the same, we have found it impossible to distinguish the influence of any factor if they are simultaneously affected. We believe that overgrowth of the bottom by filamentous algae in Listvennichny Bay (Site 2) is due to anthropogenic influence. If the development of filamentous algae was the only response to climate warming, we would also observe a similar pattern of bottom overgrowth in the background area (Site 1). However, this is not the case at Site 1. Apparently, the additional influx of nutrients to the coastal zone of Listvennichny Bay (Site 2) is through groundwater (Table 1) because of the high recreational load on its shores. A similar picture is observed in other areas of Lake Baikal [18]. We assume that climate warming only intensifies the processes of eutrophication in such areas.

Although Lake Baikal is a deep ancient water body, the observed processes in its coastal zone have similar features to those in numerous lakes of different latitudes. The approach we propose to identify communities of aquatic organisms on the basis of dominant taxa biomass as well as key species can be applied to other water bodies to understand the current and future status of biodiversity under conditions of climate change and anthropogenic impact.

## 6. Conclusions

The main autotrophic components (phytoplankton, benthic macro- and microalgae), as well as heterotrophic zooplankton and macrozoobenthos of Lake Baikal, have exhibited a response to large-scale climate anomalies in the 2000s and to the anthropogenic impact. The response scenario of the biota in the coastal zone of Lake Baikal mostly coincides with scenarios of other lakes in the world and reflects common natural patterns (high trophic level, increased role of small-sized organisms, and development of benthic filamentous algae in areas under an increased nutrient load). The key species in communities of Baikal aquatic organisms have become representative cosmopolitans and are widespread in the Palearctic. The endemic species have tended to reduce their contribution to the formation of the total biomass of plankton and benthos in Lake Baikal. Previously, most open-shore areas of Lake Baikal (including Listvennichny Bay) were considered oligotrophic, with only deep inland bays and the sandbars (aquatic areas separated from the lake by a ridge of sand) in the central and northern basins of Lake Baikal being mesotrophic. At the present time, the boundaries of the mesosaprobic zone have expanded in Lake Baikal. On the basis of our study, we propose that in the future, the eutrophication zones in different areas of Lake Baikal, which are currently confined to settlements and the outflow of large rivers, bays, and the Maloe More Strait, will close, and the current oligotrophic areas of the Baikal open coasts will become eutrophic. The anthropogenic component has intensified the processes of eutrophication of freshwater bodies under conditions of global climate change. Human modification of shorelines potentially increases trophicity, leading to shifts in the community structure of the coastal zone of Lake Baikal. Particular attention should be paid to the dynamics of the identified key species. Rearrangement of species composition will affect the stability of trophic relationships formed in the historical past periods. Further study is required to understand food webs and interspecific interactions in Lake Baikal, as well as to assess the consequences of ongoing changes in the structure of recent planktonic and benthic communities, in order to reveal the direction of processes associated with large-scale gradients spanning near the shore, off the shore, and in deep-water environments. Monitoring and management plans of the Baikal ecosystem to improve eutrophication control and mitigate natural-climatic factors should be necessary. At this stage of the study, the stability of the communities of dominant species over time is not yet clear, especially in plankton (as their boundaries are blurred in space) and benthos. To clarify this question, it is necessary to conduct further studies using the approach proposed in our work at the same stations with the specified coordinates and at the same time period. The application of saprobity indices also characterises the direction of the processes occurring in the coastal zone towards eutrophication. However, their absolute values have not yet reached the maximum; nevertheless, we can say that the communities in Listvennichny Bay are at the initial stage of anthropogenic succession. It is of interest how and at what rate they will change over time to understand the consequences of changing biodiversity and assess its stability both locality and regionally. Despite the specificity of climatic conditions in different regions for making informed decisions on biodiversity management aimed at its conservation, the results obtained can serve as a basis for assessing the rate of its change over time.

## Figures and Tables

**Table 1:** Concentrations of nutrients (mg L^−1^) in the water of the study area in August 2016. Site 1 included stations I, II, and III in the background area; Site 2 included stations IV, V, and VI in the area with human activity in Listvennichny Bay; TDP—total dissolved phosphorus; TP—total phosphorus.

Area	Water	Si	NO_2_^−^	NH_4_^+^	NO_3_^−^	P-PO_4_^3−^	TDP	TP
Site 1	surface water of Baikal coastal zone	0.23 ± 0.03	0.003 ± 0.002	0.009 ± 0.000	0.10 ± 0.00	0.004 ± 0.001	0.006 ± 0.001	0.018 ± 0.009
	near-bottom water of Baikal coastal zone	0.18 ± 0.03	0.002 ± 0.001	0.009 ± 0.003	0.14 ± 0.02	0.004 ± 0.000	0.006 ± 0.002	0.010 ± 0.001
	groundwater of Baikal beach	2.71 ± 2.50	0.006 ± 0.004	0.015 ± 0.005	0.23 ± 0.04	0.005 ± 0.001	0.013 ± 0.007	0.025 ± 0.014
Site 2	surface water of Baikal coastal zone	0.19 ± 0.01	0.002 ± 0.001	0.008 ± 0.001	0.11 ± 0.00	0.003 ± 0.001	0.005 ± 0.000	0.010 ± 0.001
	near-bottom water of Baikal coastal zone	0.17 ± 0.02	<0.002	0.007 ± 0.001	0.14 ± 0.02	0.003 ± 0.001	0.005 ± 0.000	0.011 ± 0.001
	groundwater of Baikal beach	1.37 ± 0.64	0.034 ± 0.026	0.009 ± 0.001	0.23 ± 0.04	0.030 ± 0.006	0.035 ± 0.005	0.063 ± 0.011
Rivers	mouth of the Krestovka and Cheremshanka rivers	7.07 ± 0.22	0.038 ± 0.033	0.009 ± 0.003	3.94 ± 3.09	0.027 ± 0.018	0.029 ± 0.018	0.034 ± 0.019
RD	surface water of Baikal coastal zone near Berezovy Cape in 2000 according to [26]	0.46	0.002	0.010	0.08	0.002		
	near-bottom water of Baikal coastal zone near Berezovy Cape in 2000 according to [26]	0.47	0.007	0.035	0.23	0.004		
RD	surface water of Baikal southern basin in the 1987 according to [27]	1.14			0.23	0.004		

**Table 2:** Index of biotope confinement of dominant species of aquatic organisms to the studied areas in the coastal zone of Lake Baikal: RD—reference data, Site 1—background area, Site 2—area with human activity in Listvennichny Bay; the maximum positive *F_ij_* values are in bold.

Taxa	*F_ij_*		
	RD	Site 1	Site 2
Phytoplankton			
*Anabaena lemmermannii* P. Richt.	−0.316	**+0.651**	−0.589
*Asterionella formosa* Hass.	−0.996	+0.284	+0.331
*Ceratium hirundinella* (O. Müll.) Schrank.	−0.633	+0.267	+0.182
*Dinobryon cylindricum* Imhof.	−0.999	+0.179	+0.427
*Dinobryon divergens* Imhof.	−0.988	+0.185	+0.419
*Dinobryon sociale* Ehr.	−0.985	−0.236	**+0.701**
*Gymnodinium coeruleum* Ant.	+0.120	−0.135	0.000
*Rhodomonas pusilla* (Bachm.) Javorn.	**+0.529**	−0.222	−0.451
*Spirogyra* sp.	−1.000	+0.010	**+0.557**
*Fragilaria radians* Kütz.	−0.988	+0.202	+0.403
picoplankton	**+0.735**	−0.539	−0.591
Zooplankton			
*Asplanchna priodonta* Gosse.	−0.809	**+0.551**	+0.441
*Bosmina longirostris* (O.F.Müller).	−0.693	+0.417	+0.497
*Cyclops kolensis* Lilljeborg.	**+0.880**	−0.909	−0.403
*Daphnia galeata* Sars.	+0.184	−0.324	+0.368
*Epischura baikalensis* Sars.	+0.047	+0.057	−0.644
*Leptodora kindtii* (Focke).	−1.000	**+0.714**	+0.423
*Synchaeta grandis* Zacharias.	−0.928	+0.370	**+0.712**
Benthic macroalgae			
*Spirogyra* sp.	−1.000	−1.000	**+0.999**
*Chaetocladiella pumila* (Meyer) C.Meyer et Skabitsch.	+0.401	**+0.306**	−0.974
*Cladophora floccosa* C.Meyer.	**+0.880**	−0.779	−0.850
*Draparnaldioides baicalensis* C.Meyer et Skabitsch.	+0.301	**+0.418**	−1.000
*Draparnaldioides pilosa* C.Meyer et Skbitsch.	−1.000	−1.000	**+1.000**
*Tetraspora cylindrica* var. *bullosa* C. Meyer.	**+1.000**	−1.000	−1.000
*Schizothrix* sp.	**+0.601**	+0.060	−1.000
Benthic microalgae			
*Cocconeis placentula* Ehrenb.	−0.853	**+0.453**	**+0.649**
*Cymatopleura solea* (Bréb) W.Sm.	+0.206	+0.343	−1.000
*Didymosphenia grunowii* Lange-Betalot & Metzel.	−0.131	−0.311	+0.320
*Encyonema pseudoturgidum* Pomazk. & Rodion.	**+1.000**	−1.000	−1.000
*Hannaea baicalensis* Genk., Popovsk. and Kulik.	**+0.987**	−0.984	−0.983
*Nitzschia dissipata* (Kütz.) Grun.	+0.491	−0.366	−0.480
*Nitzschia recta* Hantzsch.	**+1.000**	−1.000	−1.000
*Yasnitskya tatyanae* Pomazk. & Rodion.	−1.000	−1.000	**+1.000**

**Table 3:** The trophic level of the study area in the coastal zone of Lake Baikal (August 2016): RD—reference data, Site 1—background area, Site 2—area with human activity in Listvennichny Bay; *—ratio of flagellates biomass to endemic species biomass; B—average biomass (mg) for phyto- and zooplankton per m^3^, for macro- and microalgae per m^2^; m—standard error.

Value	Phytoplankton			Zooplankton			Macroalgae (Meyo-, Macrophytes)			Microalgae		
	RD	Site 1	Site 2	RD	Site 1	Site 2	RD	Site 1	Site 2	RD	Site 1	Site 2
Number of taxa	42	45	46	26	19	24	15	23	28	35	33	33
β-diversity by Shannon, H	1.5	2.8	2.7	1.2	1.4	2.0	1.8	1.0	2.1	2.0	2.1	1.7
Number of indicator species	36	37	39	18	13	17	7	10	11	27	20	18
Saprobity index, *S*	1.47	1.60	1.70	1.45	1.39	1.53	1.48	1.57	2.17	1.0	1.28	1.45
Ratio of eutrophic species number to oligotrophic species number, E/O	2:1	2:1	2:1	1:2	1:2	1:2	1:1	1:2	3:1	1:3	1:2	1:2
Ratio of mesosaprobiont species biomass to endemic species biomass, M/E	1:6 *	3:1 *	6:1 *	1:4	1:5	2:1	1:18	1:7	3:1	1:47	1:2	7:1
Total biomass, B ± m	190.7 ± 41.5	186.9 ± 22.5	246.0 ± 16.3	672.7 ± 158.6	723.7 ± 166.1	100.7 ± 14.3	245,500 ± 65,500	185,287 ± 74,870	367,564 ± 119,414	39.3 ± 10.5	10.0 ± 3.2	12.7 ± 5.3

## Data Availability

The data that support the findings of this study are available from the corresponding authors upon reasonable request.

## Abbreviations

TP	total phosphorus
TDP	total dissolved phosphorus
TC	thermotolerant coliforms
CFU	colony forming units
Tr.	transects
s	square
RD	reference data
PCA	principal component analysis
H	the Shannon diversity index
E/O	the ratio of eutrophic to oligotrophic species counts
M/E	the ratio of mesosaprobiont biomass to the biomass of Baikal endemics
*S*	the saprobity index of a water body or its part
*F_ij_*	the index of relative biotope confinement
